# ‘I had no choice!’ Challenges in the informed consent and decision-making process for allogeneic stem cell transplantation: a qualitative method triangulation

**DOI:** 10.1186/s13287-025-04766-9

**Published:** 2025-11-07

**Authors:** A. Nowak, S. Nadolny, F. Jahn, J. Schaffrath, L. P. Müller, J. Schildmann

**Affiliations:** 1 Clinical Ethics and Clinical Ethics Committee, University Hospital Schleswig-Holstein, Arnold-Heller-Straße 3, 24105 Kiel, Germany; 2 Institute for History and Ethics of Medicine, Interdisciplinary Center for Health Sciences, Medical Faculty, Martin Luther University Halle-Wittenberg, Halle (Saale), Germany; 3 Institute for Educational and Health Services Research in the Health Sector, Bielefeld University of Applied Sciences, Bielefeld, Germany; 4 Department of Internal Medicine IV (Haematology/Oncology), University Hospital Halle, Martin Luther University Halle-Wittenberg, Halle (Saale), Germany

**Keywords:** Allogeneic stem cell transplantation, Informed consent, Decision making, Advance care planning, Ethical challenges

## Abstract

**Background:**

For some patients allogeneic stem cell transplantation (alloSCT) offers the only chance for cure. There is limited empirical data on the informed consent (IC) process and respective perceptions and evaluations of alloSCT patients so far.

**Objective:**

The objective of this single centre empirical-ethical study is to explore the IC and decision-making process in the context of alloSCT at a German university hospital, with a particular focus on ethical challenges.

**Study design:**

From 10/2021 to 12/2023, 8 patients were followed during 16 separate IC consultations on alloSCT, by non-participant observation respectively. In addition, a separate interview in the absence of other medical personnel was conducted with the patients after alloSCT about the consultations and the reasons for their decision. Transcripts were analysed using the Qualitative Analysis Guide of Leuven and included triangulation of interview and observational data.

**Results:**

We observed eight patients at the two IC consultations each and interviewed seven after completed alloSCT. Consultations were carried out by three different physicians and together lasted a median of 89 min. The patient surveys were conducted at the time of the patients’ discharge from hospital, after the transplant had been performed. Patients had a positive overall impression of the information process and appreciated it. A central theme in the analysis was patients’ impression of having had “no choice” in the treatment decision. Various possible reasons for this narrative could be identified in the interviews and triangulated with the results of our observations. The patients’ motives for their choice were: the *therapeutic goal of healing*; *fear of death*; *the desire to live longer* and *trust in medical expertise*. There was often a *lack of awareness about other treatment options* on the side of the patients. Observations suggest that the consultations *focused* on the *physician’s recommendations* while palliative treatment was presented in a limited way. *Extensive planning* which is necessary for successful alloSCT *may be perceived as organisational pressure* on decision-making by patients.

**Conclusion:**

The narrative of not having had a choice is complex and should be explored further by a specific catalog of specific questions. In particular, the organisational processes and the pressure they may place on patients should be examined more closely and reflected upon.

**Supplementary Information:**

The online version contains supplementary material available at 10.1186/s13287-025-04766-9.

## Background

Allogeneic stem cell transplantation (alloSCT) is an established treatment option for patients with haematological diseases such as acute myeloid leukaemia (AML). For many patients, it is the only chance of a long-term cure for their disease and is therefore clearly recommended as standard therapy in the relevant guidelines for suitable patients. The chances of success vary depending on the disease, age and health of the patient, as well as other factors, such as compliance and adherence to therapy. There is a significant risk of therapy-associated mortality and a serious risk of a relapse of the underlying malignant disease. At the same time, patients may encounter severe physical and psychological stress during conditioning [1–3]. A wide range of side effects and life-threatening risks can remain even after the transplant, in addition to potentially life-altering restrictions on daily life. The latter and the associated impairment at work or stress in relationships with relatives can also lead to severe psychological stress for patients [4].

The decision about an alloSCT, which often represents the only chance for cure, on the one hand, and the life-threatening risks and everyday restrictions on the other, is, thus, associated with great uncertainty for decision making regarding the benefits for and harms to the patient. The normative framework for the decision-making process is the informed consent (IC) [5]. Due to the complexity of alloSCT and the multifactorial high-risk character associated with the vulnerability of patients as a result of being confronted with their terminal illness, there is a discussion in the literature as to whether it is actually possible to elicit full IC in the context of alloSCT [6–9]. Furthermore, recommendations were made to improve the IC prior to alloSCT [10–14].

Only limited empirical data on the IC process and the perceptions and evaluations of alloSCT patients regarding the decision-making process have been published so far. Such data are necessary to compare the normative requirements of IC and current practice. In addition, such data provide a starting point for empirical-ethical analyses regarding further developing the design of IC process prior to alloSCT. The object of this study is to explore the IC and decision-making process in the context of alloSCT at a German university hospital, with a particular focus on ethical challenges.

## Methods

The study was conducted as a qualitative case study with a methodological triangulation of non-participatory observation and qualitative interviews with the stakeholders involved in the alloSCT.

### Qualitative case study

The qualitative case study is used to explore a phenomenon or situation in a real-life context [15]. The ‘case’ was defined as adults with a haematological disease and an indication for an alloSCT in their IC and decision-making process at the haematological-oncological department of a university hospital, as well as their physicians and healthcare professionals involved in the IC process.

### Data collection

Data was collected using a triangulation of methods from non-participant observation of the IC consultations (T1 and T2) and semi-structured, problem-centred interviews [16] with the physicians providing the information, and the patients (T3) and nurses involved concerning the process and challenges of the IC and decision-making process in the context of alloSCT. The IC consultations and interviews were audio-digitally recorded, then transcribed and anonymised. Defined aspects of the discussions were also recorded using an observation protocol.

### Recruitment

The recruitment of participating patients was carried out on the basis of a pre-selection conducted by the leading senior physician. We chose the senior physician as gatekeeper, as he has an overview of all patients in the department and was able to assess them based on the aim of the study. Furthermore, the study was intended to provide insight into the typical information processes on the ward. It was therefore necessary for the senior physician to preselect typical patients. Due to the vulnerability of the patient group, additional confrontations between emotionally distressed patients and external researchers should be avoided. Potential participants were informed orally about the possibility of participating in the study and, if they were generally willing to participate, they received an information letter and the consent form. If they were interested, a detailed IC discussion was conducted by the observing research team without the presence of members of the treatment team. Thus, pressure on patients to participate in the study should be avoided. Three physicians at the department provided information about alloSCT. All of them were proactively approached by the research team, and if they were interested, they received detailed information. The recruitment of nurses and psychologists followed the same procedure.

### Sampling

The selection of participants was carried out by means of theoretical sampling. The basis for the further selection of participants was an interest in the findings and the filling of previous gaps, as well as the identification of deviating or complementary cases to the existing data material. The decision on the sample size was made taking into account the theoretical saturation.

### Data analysis

The data analysis of the transcripts was carried out using the methods of Grounded Theory [17]. The Qualitative Analysis Guide of Leuven (QUAGOL) [18] was used to structure the analysis process. The data was analysed using a case-based and cross-case approach. A multi-perspective, detailed case description was created for each case. The data was coded using open, axial and selective coding [17]. The data was first analysed by the observer (AN) and two other researchers (JSchi and SN) with backgrounds in medical ethics, medical law, haematology and nursing sciences who were not employed at the studied department. The results were then discussed in five internal quality circles with researchers with no relationship to the department. In a further step, the researchers discussed anonymised key statements made by patients and their interpretations “in the sense of member check”. Comments and questions identified jointly with the observed physicians were then used for further analysis by the first author.

The study was approved by the ethics commission of the medical faculty of the Martin Luther University Halle-Wittenberg (processing number: 2020-007).

## Results

### Observations and interviews

This paper analyses evaluations of transcribed consultations between physician and patient, as well as interviews conducted by the researcher with the patients. During the study period, potential participants were informed about the study by the gatekeeper. Nine patients expressed interest. Of these patients one patient approached by the first author declined to participate due to fears that the interview would be emotionally stressful. One patient died during therapy. The patients included in the study represent a ‘typical’ patient at the department under investigation. All were Caucasian. Seven of eight patients had AML/MDS as their underlying disease, which is the main reason for alloSCT in the region [19]. Table 1 summarises the socio-demographic characteristics of the patients. All consultations were conducted by experienced physicians with an average of 17 years’ experience in haematology and oncology. In accordance with the respective guidelines, alloSCT comprised a standard indication for the patients’ diagnoses.

**Table 1:** Overview of the socio-demographic data of the patients*

Patient	Sex	Age group (range of 10 years)	Disease	Status 1 year after transplant	Relatives present at consultation?
P1	m	60–70	Secondary AML^1^ from MDS^2^	Deceased (GvHD^4^)	No
P2	w	30–40	Philadelphia-positive ALL^3^	NED^5^	Yes
P3	m	50–60	Secondary AML from MDS (relapse of initial disease)	NED	Yes
P4	w	60–70	AML	Deceased (relapse)	Yes
P5	m	40–50	AML (molecular relapse of initial disease)	NED (severe GVHD)	No
P6	m	60–70	Hypoplastic MDS	Deceased (pneumonia)	No
P7	m	60–70	Hypoplastic MDS	NED	No
P8	m	60–70	Secondary AML from MDS	Deceased (MODS^6^)	No

^1^Acute myeloid leukemia; ^2^Myelodysplastic Neoplasms; ^3^Acute lymphoblastic leukemia; ^4^Graft-versus-host disease; ^5^No evidence of disease; ^6^Multi organ dysfunction syndrome

The IC process was divided into two consultations (C1 and C2). With many patients, conversations about alloSCT had already taken place prior to the IC consultations observed, for example during previous inpatient stays or during previous treatments for their disease. In addition, the patients had given their consent for a donor search. The entire IC process observed in this study (C1 + C2) took an average of 89 min (range: 67–110 min). Relatives were present during the process for *n* = 3 patients. All patients underwent oncological therapies, ongoing examinations and an interdisciplinary discussion between diagnosis and C1 to determine their suitability for an alloSCT. C1 lasted an average of 61 min (range: 29–93 min). C1 was conducted using an internal information sheet, which was tailored to the individual patient during the consultation (contents of the information sheet are included in the appendix). The document was given to the patient to take home. To supplement the information, comprehensive information material from the clinic and DKMS (https://mediacenter.dkms.de/wp-content/uploads/2023/04/Der-Rote-Ratgeber-Band-1.pdf?s=393331) was also provided. In this article, the transcribed consultations were evaluated. C2 took place after admission to the hospital, 24 h before the planned start of conditioning treatment. An average of 23 days (range: 6–39 days) had passed between C1 and C2. While C1 was conducted in a standardised manner using the specified information sheet and differed only in the detail of the discussion, C2 varied in both content and scope. The content of C2 ranged from repeating large parts of the information provided in C1, including a detailed discussion of the current treatment plan, to discussing interim changes to the plan and the planned course of treatment, to only asking whether there were any questions and announcing that treatment would then begin as discussed. Table 2 provides an overview of the number and duration of the IC consultations observed and the duration of the patient interviews.

**Table 2:** Duration of the IC consultations observed and the interview

Patient	Duration of IC process (C1 + C2) in minutes	Duration of C1 in minutes	Duration of C2 in minutes	Duration between C1 and C2 in days	Duration of interview in minutes
P1	95	54	41	22	26
P2	95	50	45	23	27
P3	95	63	32	39	38
P4	77	53	24	23	50
P5	110	93	17	26	28
P6	89	79	10	6	25
P7	86	68	18	17	32
P8	67	29	38	24	n.a.
Average / Median (R: min-max)	∅: 89±13 /Md: 92 (67–110)	61±19,5 /Md: 58,5 (29–93)	28±12,7 /Md: 28 (10–45)	22,5±9,2/ Md 23 (6–39)	32±9,9 /Md 28 (25–50)

### Content analysis

The presentation of the results is based on the evaluation of the interviews with the patients and the observations of the IC consultations. The quotations serve as evidence for the data analysis as typical examples for narratives of research participants. In the course of the analysis, the categories ‘evaluation of the IC-consultation’, ‘reasons for decision-making from the patient’s point of view’ and ‘organisational factors’ were developed. Each category encompasses several subcategories.

#### I. Evaluations of the IC-consultation – ‘I was completely satisfied with everything’ – few questions and questionable understanding

All patients interviewed expressed themselves positively regarding the IC process, the physicians and the treatment team. The patients particularly emphasised the time the clinicians took to explain the therapy, as well as the honesty, empathy and calmness they perceived. They felt well-informed and emphasised that they could ask questions at any time and always received answers that were tailored to their needs.

I have to say that I was completely satisfied with everything. Yes, it was just like with nurses and physicians, the information, I can’t say anything negative.

P1 Interview: 23–24

I got a comprehensible answer to every question I asked. One that I understood, not technical jargon. So everything is fine; I’m totally satisfied.

P7 Interview: 20–22

##### Triangulation of interview and observational data

It was noticeable during the observations that the patients asked very few questions during the IC consultations. The questions that were asked mostly revolved around the need for information regarding the start and duration of the treatment, as well as the possibility of receiving visitors.

#### II. Reasons for the decision from the patient’s point of view – ‘I had no choice’, focus on the possible benefits of SCT

The narrative that dominated among patients during the interviews was that they had no choice or did not make any decision about therapy at all.

I had no say in the matter. There was no alternative. I could have jumped on the ceiling or said I didn’t want it, but that wouldn’t have changed anything.

P3 Interview: 31–32

Finally, I had no choice. The only chance left was to have the allogeneic stem cell transplant.

P7 Interview: 16–17

The interviews and observations identified a range of factors, individual and organisational, that could have led to the perception that patients had no choice in the decision. It is not always possible to draw a clear line between the influencing factors.

##### The goal is the healing – no realistic curative alternative

In the case of three patients, the chance of healing was identified as an important guiding motive for the decision to conduct the alloSCT. Due to a lack of curative alternatives or the high chances of a relapse with other therapy options, the alloSCT was said to be the only choice to achieve this goal.

Yes, as I said, the tendency is that you want to take the safest route for yourself. The one that promises the most success in healing.

P2 Interview: 82–83

Here, the individual assessment of the patient was in the centre of attention, that the desire for healing left them no choice but to opt for alloSCT.

##### Fear of dying and the desire to prolong life

Fear of dying and the will to live (longer) could also be identified as individual motives for the decision.

Yes, the crucial point was just that my life would be extended as a result. […] That’s it. I still want to live a few more years, it’s too early for me to leave this world.

P1 Interview: 37–38

In view of the patients’ statements, it was noticeable that the decision for alloSCT was equated with one in favour of life and a decision against transplantation with the onset of (rapid) death.

Yes, but there was no other way for me to live. Otherwise I would have died for sure. Without treatment you die, don’t you?

P6 Interview: 44–46

##### Lack of knowledge about or not engaging with alternatives

Although the patients’ statements about the lack of treatment alternatives could also be interpreted as their own assessment of their chances of recovery or long-term survival, the analysis of the interviews also showed that six of the seven patients interviewed had little or no knowledge of the ways of proceeding with palliative treatment strategies or neglected them in the light of a curative therapy option and their will to survive.

I don’t know what the other alternative would have been (…), there wasn’t one. So, it was just a case of, yes, okay, I have to do this now and I have to get through it. There was no other decision to choose from.

P4 Interview: 16–19

##### Trust in medical expertise/transfer of decision to the physician

Furthermore, the evaluation showed a conscious or unconscious transfer of decision-making authority to the treatment team. In some cases, the treatment team’s recommendations before or during the consultation were interpreted as the best personal decision because of the belief in medical expertise.

Well, I studied something different, so I don’t have a medical degree. In this respect, you can only trust those who have one and are skilled in it. […] and that’s why there was no questioning of the decision […].

P4 Interview: 79–84

##### Triangulation of interview and observational data

It was noticeable while observing the conversations during the IC consultations that the recommendations expressed by the physicians repeatedly included suggestive formulations.

[…] and that’s why we strongly recommend the transplant.

P1 C2: 106

[…] I urgently recommend it to you, it is the most optimised plan.

P2 C1: 351–352

Treatment alternatives were presented in all IC processes. In addition, the observations suggest that the treatment alternatives to alloSCT were presented in a shorter form and with a focus on the chances of recovery.

A: The alternative would be to continue the chemotherapy […] that would be three more cycles, where you would be in hospital for three, well, two to four weeks each time. And that would be done three more times. And then they would just monitor you, puncturing your bone marrow again, regularly. But that would be the end of the therapy, and as I said, the chances of recovery are around twenty per cent.

P: Well, okay. I’d much rather have the higher chance of recovery.

A: That’s why we clearly recommend it. Another alternative is certainly to do nothing, but I’m not going to write that here. In my view, at your age and state of health, that’s not an alternative.

P1 C1: 181–193

In our observations, no comprehensive discussion of alternatives was observed in any of the IC consultations. It is beyond the method to state whether such discussions have taken place elsewhere.

#### III. Organisational factors – ‘It was clear from the start’

***‘That was too fast for my liking’***

There are repeated descriptions of a tightly scheduled and planned course of treatment in the patients’ statements, which could give the impression that no decision or consent is necessary.

I don’t even know if I made the [decision] at all. I got the diagnosis at the beginning of May […]. It was all so helter-skelter […] Well, and then […] I signed that we were looking for a donor. But it was worded as if it could be that we needed a donor, we don’t know yet. At least, that’s how I understood it. […] So, after the first hospital stay, I was down in the outpatient clinic and the doctor [Wegner*] came right over and said, yes, we have already found three donors. […] And then I thought, […] then I no longer need to decide. The decision had already been made. So, that was too fast for my liking. There is no going back then, when suddenly the donor is already at the door. But well, now it has turned out that way.

P4 Interview: 344–373; *Pseudonym

***‘That was clear right from the start’***

Furthermore, the patients stated that they had already made a decision in favour of the transplant before the IC consultations took place. This was usually based on the diagnosis discussion (which were not observed in the context of this study), although at that point it was not yet clear whether they would be considered for a transplant. These discussions were characterised in the patients’ narrative by an affirmative tone of language.

That was clear from the outset, because I was the first to talk to Doctor [Davids*]. I went […] to Doctor [Davids*] and she told me from the outset, Mr [P1], you can do it! Yes, well, she also explained to me what it was all about and what it involves, and she encouraged me and said, ‘You can do it!’ Without any problems. And I said, well, it certainly doesn’t go off without a hitch! No, she said, but there won’t be any major difficulties. It’s quite normal for side effects to occur. It’s the same with every illness […].

P1 Interview: 89–96; *Pseudonym

Regarding their decision, some patients drew a very passive picture of themselves, whereby the illustration appears as if they were an object of medical treatment decisions and not decision-makers themselves. At the same time, this also seemed to relieve some patients.

It was more of a burden lifted. It’s good to know that you don’t have to make a decision.

P3 Interview: 169–170

The physicians chose this path. Maybe you should ask them first. [laughs]

P7 Interview: 347–348

##### Triangulation of interview and observational data

The observations in C1 already showed a far-reaching and detailed planning of the process, starting with the date of admission, continuing with the date of transplantation and ending with the date of discharge and follow-up appointments.

This is the plan. […] [W]e have now planned the transplant for December [XX] or [XX], according to the protocol. That means you won’t be home for Christmas. […] [T]hat means that if everything goes well […] you’ll be home around mid-January. […] We’ll give you taxi vouchers, and then you’ll come to our outpatient clinic once a week […]. After thirty days, […] then again on days 60 and 120 and then every three months. […] And then we would give you these cells again on an outpatient basis. From day 150, so five months, six months after the transplant.

P2 C1: 293–573

At the same time, this plan and the patient’s risk profile could change as a result of the interim check-ups until shortly before the start of the therapy. It is to be clarified whether a reflection on these changes by the patient is possible at the time of the IC consultations, after admission to the ward and 24 h before the start of the therapy.

You have this limited lung function, which is why the risk – back then I said fourteen per cent – is now […] between twenty and twenty-five per cent.

P1 C2: 377–380

**Fig. 1:**
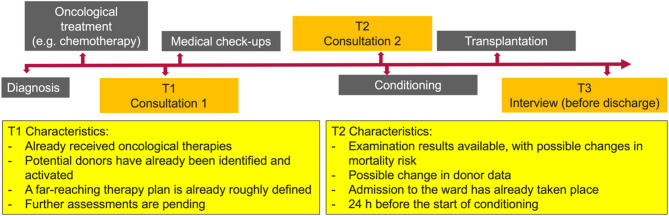
Characteristics of the timing of the consultations

**Fig. 2:**
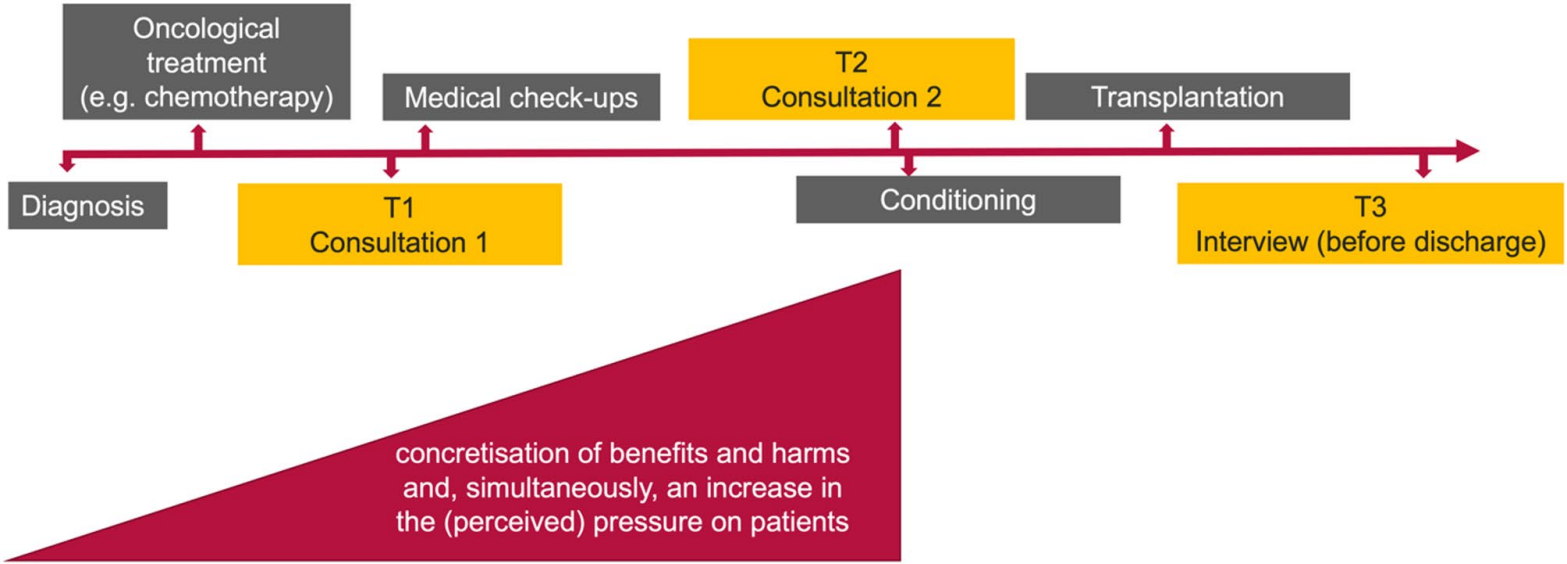
Risk of reduced perceptible freedom in decision-making

Figures 1 and 2 illustrate the characteristics of the IC process and show the risk of how organisational and communicative processes are perceived by patients as pressure or reductions in their freedom in decision making.

## Discussion

Our study is, to the best of our knowledge, the first qualitative study in Germany to focus on the ethically relevant perception of the information process and decision-making of patients prior to alloSCT by triangulating non-participant observations of the IC consultations on alloSCT with the interviews of the relevant stakeholders. With regard to all findings and their interpretation it is important to acknowledge that due to the method used these cannot be generalised. A first finding is the general positive perception of the IC consultations and valued esteem of the patient situation by the respective medical personnel. C1 and C2 combined, the average duration of IC conversations per patient was 89 min. This duration is clearly longer than the 40–60 min described in the literature [12, 20]. This may also have contributed to the positive perception of the discussions by the patients. Secondly however, the patients reported the subjective impression of a lack of choice from their perspective and relevant putative factors contributing to this perception were identified. A third result, which is also relevant for the design of the information process, is the description of organisational framework conditions that potentially restrict the perception of decision-making options.

The narrative of having had no choice regarding the decision can also be found in the international literature [21–23], but especially as a side topic. In principle, this statement can be seen as problematic, as the IC requires patients to make a free decision in favour of or against the proposed treatment [5]. However, our exploration of this patient narrative also draws attention to the ambiguity of the statement. Our study was able to identify several specific motives behind this narrative, such as unwavering hope of a cure thus perceiving the lack of curative alternatives as having no alternative or trust in medical expertise. These motives, on the other hand, are completely in line with the requirements of an IC, because they are based on a free decision by the patient [22]. At the same time, the triangulation of the stakeholder interviews with our observations offers opportunities for a critical reflection on the motives. Even if the patients felt very well-informed, the lack of awareness on palliative treatment options at the time of post-SCT interview raises the question of how valid this impression is. There is a difference between whether the motivation for performing an alloSCT is the desire for healing or the fear of death. Although alloSCT is often the only chance of healing, it also involves the risk that patients may die earlier as a result of the treatment than if they had opted for palliative care. Cooperation with palliative care teams could lead to a more comprehensive presentation of treatment alternatives [24–26]. However, it could also represent a form of post-SCT neglect of other options than the chosen one or specific neglect of non-curative and thus more terrifying options. Our findings in the evaluation of the narrative of having no choice in the therapeutic decision to undergo alloSCT resemble in various respects with the study by Unsöld et al. from [27]. Differences between the studies are the patient group (patients with lung cancer and limited life expectancy) and the chosen methods. Compared to this study, the strength of our study is the comparison of the interviews with the triangulation of the results with observed practice.

Comparable to findings from other studies [21, 23, 28], it was also clear in our study that most patients had already made their decision to undergo alloSCT before the first treatment counselling interview. The treatment consultation could therefore serve as an opportunity to jointly validate this decision, which was made in advance and may still be “uninformed”. What is new about our study, however, is that with regard to the studied investigated group of patients we were able to identify clear signs that due to the close planning of the therapy, patients may get the impression that they have no choice right from the start, even when confronted with their disease, because the treatment scheme has already been decided upon by physicians. Such an impression can be reinforced by the necessary extensive and detailed planning of this complex process, for example, by identifying donors before the first information session or presenting a detailed plan of the treatment process. While other studies have identified pressure from families in particular [22], our study at least with regard to the researched sample rather points to the risk to perceived pressure from the organisational processes. These results could also support the criticism of Jordens et al. [9], who pointed out the danger of tacit consent in the context of IC processes in which the patient’s appearance or signing of a form (i.e. here the consent for donor search) implicitly interprets the treatment team’s understanding and considered consent to the entire treatment. Developing trust between the patient and the treatment team [29] alone could even reinforce this dynamic. Instead, it seems ethically necessary to make the reasons for the patient’s choice explicit in a joint discussion in order to address any misunderstandings or gaps in knowledge. This could minimise the risk of patients undergoing alloSCT due to the systemic pressure inherent in treatment planning or due to a lack of knowledge about (palliative) treatment alternatives. One possibility for this could be to divide the first long consultation into two shorter ones, with the second consultation accompanied to ensure understanding and reflection questions [12]. Following from these analyses it seems firstly necessary to conduct more research addressing the relevance, causes and impact of the reported issues. Specifically, the following hypotheses should be addressed using the respective methods:

1. Choiceless situation right from the start? Design: Subcohorts with one interviewed at time of initiating donor search, one before and one after first IC conversation, one before and one after second IC conversation and in all groups observation of the respective preceding conversation (i.e. 5 groups).
2. Actually choiceless or perceived choiceless? Design: Interviews as for 1.) and including explicit questions on specific knowledge of alternatives, risks of SCT etc.
3. When and why do patients decide against a transplant during the process? Interviews with patients who decided against a transplant despite the recommendation of the treatment team. Interviews with treatment teams on their perception of differences in the information process for these patients.

Yet, based solely on the results of this qualitative single center study the options to minimise the perceived pressure for patients should be tentatively explored. Secondly, medical communication training and the use of decision aids or advance care planning concepts on the side of the informing medical personnel could contribute to an improved perception of treatment alternatives [13, 20, 30–33]. The use of patient-reported outcomes or easy-to-read information sheets showing outcomes as percentages could also be helpful in this regard [34]. A comprehensive investigation of these instruments in alloSCT is pending [6].

## Limitations

This study is a monocentric qualitative evaluation of informed consent discussions and decision-making processes in a very small number of patients. The significance of our study is, therefore, limited, the results are not representative and must be viewed with particular caution. The interview analysing the perception of the patient is performed after the alloSCT. This may carry a bias, as the patients have undergone the procedure perceived being without alternative. Psychological repression of the maybe more frightening less hopeful option can not be excluded. The qualitative analysis of the interviews and observations can also be biased when interpreted by researchers. The first author (AN) was both the observer and the interviewer. In order to validate the evaluation and interpretation, uninvolved researchers were, therefore, also consulted as part of five quality circles. The fact that the physicians who provided the information participated in the evaluation of the results may also have led to biases. This was counteracted in the research process by the primary evaluation being carried out by researchers who were not involved in the IC process and by the constant comparison of all interpretations with the data material. The position of the leading senior physician as gatekeeper may also have led to biases, as patients may have perceived this as pressure to participate in the study or to give supposedly desired answers. The researchers counteracted this by conducting the information and consent discussions for the study without the members of the treatment team being present and by discussing the patients’ statements with the physicians who provided the informed consent for the treatment only in excerpts and in anonymised form.

## Supplementary Information

Below is the link to the electronic supplementary material.

Supplementary Material 1.

## Data Availability

The datasets generated and/or analysed during the current study are not publicly available due to confidentiality reasons but are available from the corresponding author on request.
