# Supplementary material for: ‘I had no choice!’ Challenges in the informed consent and decision-making process for allogeneic stem cell transplantation: a qualitative method triangulation
Source: Stem Cell Res Ther. 2025 Nov 7;16:623. doi: 10.1186/s13287-025-04766-9 (PMC12593839; doi:10.1186/s13287-025-04766-9)
Supplement: Supplementary file 1 — Supplementary Material 1. [file 13287_2025_4766_MOESM1_ESM.docx]

Supplement: Contents of the consent form

| 1. Background information on the implementation of SZT   a. Current illness  b. Role of the immune response  c. Alternative therapies with prospects of success and risks   1. Transplantation procedure (general) 2. Necessary check-ups 3. Information on the central venous catheter 4. Conditioning therapy   a. Procedure  b. Planned individual conditioning therapy  c. Information on side effects of medication and radiation   1. Immunosuppression and its risks   a. Necessity of immunosuppression  b. Medication and its side effects   1. Transplant   a. Planned transplant and HLA identity  b. CMV constellation and associated risk  c. Gender and blood group constellation and associated risk   1. Side effects and risks of transplantation   a. Therapy-associated mortality risk  b. Rejection reaction – transplant failure  c. Transplant-versus-host disease  d. Infections  e. Impaired bone marrow function and transmission of blood products  f. Fertility / infertility  g. Secondary tumours  h. Intensive care measures  i. Significant side effects   1. Post-treatment care   a. Inpatient phase  b. Outpatient care  c. Procedure in case of recurrence (DLI)  d. Documentation of data and scientific evaluation   1. Appeal for communication with relatives   a. Psychological stress  b. Advance directive and conversations with close relatives  11. Further information  a. Stem cell donor contact (When and how is it possible?)  b. Reference information brochures |
| --- |
